# Metformin Mitigates Cartilage Degradation by Activating AMPK/SIRT1-Mediated Autophagy in a Mouse Osteoarthritis Model

**DOI:** 10.3389/fphar.2020.01114

**Published:** 2020-07-24

**Authors:** Chenzhong Wang, Zhenjun Yao, Yueqi Zhang, Yi Yang, Jinyu Liu, Yi Shi, Chi Zhang

**Affiliations:** ^1^ Department of Orthopedic Surgery, Zhongshan Hospital, Fudan University, Shanghai, China; ^2^ Biomedical Research Centre, Zhongshan Hospital, Fudan University, Shanghai, China

**Keywords:** osteoarthritis, cartilage, metformin, autophagy, apoptosis, 5’ AMP-activated protein kinase, silent mating type information regulation 2 homolog 1

## Abstract

Chondrocyte dysfunction is a key mechanism underlying osteoarthritis. Metformin has shown protective effects in many diseases. The present study aimed to investigate the effects of metformin on autophagy and apoptosis in the process of osteoarthritis. A mouse osteoarthritis model was set up by surgically destabilizing medial meniscus in the knee. Intraarticular injection of metformin or vehicle was applied in the right knee for eight weeks. Mouse articular chondrocytes were isolated and passaged for *in vitro* experiments. Small interfering RNA (siRNA) transfection was used to silence target genes. Western blotting, immunohistochemistry, transmission electron microscopy were used. After eight weeks, metformin restored surgery-induced upregulation of MMP13 and downregulation of type II collagen in the joint cartilage. In cultured primary murine chondrocytes, IL-1β aggravated apoptosis and catabolic response in a dose-dependent manner. In the presence of IL-1β, metformin increased phosphorylated levels of AMPKα and upregulated SIRT1 protein expression, leading to an increase in autophagy as well as a decrease in catabolism and apoptosis. Inactivating AMPKα or inhibiting SIRT1 prevented the augmented autophagy in the presence of metformin. Silencing AMPKα2, but not AMPKα1, reduced SIRT1 expression and downregulated autophagy in cultured chondrocytes. Metformin protects against IL-1β-induced extracellular matrix (ECM) degradation in cultured chondrocytes and in mouse osteoarthritis model through activating AMPKα/SIRT1 signaling. Metformin shed light on the treatment of osteoarthritis.

## Introduction

Osteoarthritis (OA) is a common joint disease characterized by cartilage breakdown and joint tissue deformity (Loeser et al., 2012). Both elevated levels of inflammation and increased expressions of metalloproteinases take part in reducing extracellular matrix of cartilage in the process of OA. Chondrocytes, the primary cell in healthy cartilage, produce cartilaginous matrix, mainly composed of type II collagen and proteoglycans. However, they also produce metalloproteinases to degrade the cartilaginous matrix under disease conditions.

Balance between autophagy and apoptosis plays an important role in the progress of OA (Carames et al., 2012; McNulty et al., 2012; Musumeci et al., 2015), shown as increased levels of apoptosis (Blanco et al., 1998) and decreased levels of autophagy (Lotz and Carames, 2011) in the cartilage. Apoptosis is a programmed cell death. Increased apoptosis has been reported in OA (Hashimoto et al., 1998). Autophagy is an essential homeostatic process that maintains cell homeostasis by adjusting cell metabolism and removing damaged macromolecules and organelles. Proinflammatory cytokines inhibit autophagy, tilting the balance in favor of apoptosis (Maiuri et al., 2007). It has been reported that autophagy is impaired in the cartilage of OA patients (Carames et al., 2010), and the induction of autophagy decreases metalloproteinases expression by downregulating apoptosis (Sasaki et al., 2012). Thus, autophagy is proposed to be a promising therapeutic target for OA treatment.

Metformin is a widely used medicine in diabetes treatment (Pernicova and Korbonits, 2014). Metformin is reported to activate 5’ adenosine monophosphate-activated protein kinase (AMPK) as well as autophagy (Shi et al., 2012). In addition, metformin is well-established to activate the NAD+-dependent deacetylase silent mating type information regulation 2 homolog 1(SIRT 1) (Caton et al., 2010), which exerts protective effects on cell senescence (Hernandez-Segura et al., 2018) and the aging process (Finkel et al., 2009). The anti-aging activity of SIRT1 is achieved by fine-tuning the AMPK pathway (Ruderman et al., 2010; Wang et al., 2011). Metformin increases protein expressions of collagen-II and aggrecan in nucleus pulposus cells (Chen et al., 2016), indicating metformin has beneficial effects on the extracellular matrix (Feng et al., 2020; Li et al., 2020). Thus, the present study was designed to investigate the protective effects of metformin on autophagy in the progress of OA. Special attention was paid on AMPK/SIRT1 signaling since AMPK and SIRT1 are vital in an orchestrated network controlling cellular homeostasis and autophagy.

## Materials and Methods

### Animal Experiment

Mice experiments were approved by the Ethics Committee for Animal Research (Zhongshan Hospital, Shanghai, China). Eight-week-old C57bl/6 mice were purchased from Shanghai SLAC (SLAC Inc. Shanghai, China). Fifteen male C57BL/6 mice (n = 5 per group; 8 weeks old; mean body weight = 25 g) were randomly divided into sham group, surgical destabilization of the medial meniscus (DMM) model treated with a solvent solution [1% dimethyl sulphoxide (DMSO)]or metformin (D150959, purity: 97%, Sigma, St.Louis, USA; dissolved in DMSO). The OA model was modified based on previous reports (Glasson et al., 2007). Under anesthesia, medial meniscotibial ligament in the right knee was transected. Mice in the sham group went through the same procedure except for the transection. For intraarticular injections, the stock solution of metformin (1.65 g/ml) was diluted in PBS (1:100) and administered in the knee every three days for eight weeks (Qin et al., 2017). After eight weeks, mice were euthanized with overdose chloral hydrate. The knee joints were collected for transmission electron microscopy and histochemistry study.

### Transmission Electron Microscopy

Cartilage tissues were prepared with 5% glutaraldehyde in 0.1 M phosphate buffer (pH 7.4) for four hours at 4°C and 1% OsO4 in 0.1 M phosphate buffer (pH 7.4) for two hours at 4°C. After stepwise dehydration, the specimens were fixed with propylene oxide and embedded in Epon 812 resin. The specimens were cut into 50 nm ultrathin sections and stained with 3% uranyl acetate and lead citrate for contrast optimization. Samples were examined with transmission electron microscopy (TEM; TEM-2100, Japan).

### Histological and Immunohistochemical Analysis

The joints were fixed with 4% paraformaldehyde and embedded in paraffin. Semi-serial sections were prepared and stained with hematoxylin and eosin (HE) or Safranin-O-fast green-iron hematoxylin (Safranin O). The severity of OA was scored by OA research society international (OARSI) scoring system (Glasson et al., 2010). In brief, grade 0 indicated healthy cartilage; grade 0.5 shown a loss of Safranin O staining without structural changes; grade 1 shown small fibrillations without loss of cartilage; grade 2 shown vertical clefts down to the layer below the superficial layer; grade 3–6 shown vertical clefts or erosion to the calcified cartilage of the articular surface <25%, 25%–50%, 50%–75% and >75%, respectively. The synovial thickness of the knee joint was measured by software ImageJ (NIH). To inactivate endogenous peroxidase activity, slides were heated with citrate-EDTA buffer (pH 6.2) for ten minutes at boiling point and incubated with 3% H_2_O_2_ for ten minutes. After blocking with goat serum for one hour, slides were incubated with primary antibodies against collagen II, MMP13, p-AMPKα, SIRT1, LC3B, and cleaved caspase-3 for overnight at 4°C. After secondary antibodies were treated, the immunohistochemical signals were visualized by using DAB (Solarbio, Beijing, China). Negative control experiments were performed with corresponding isotype antibodies. Staining was analyzed quantitatively by calculating the percentage of cells staining positive for the respective biomarkers and the total number of chondrocytes in a defined 200 × 900 pixel area of articular cartilage. All quantitative analysis was performed using ImageJ (NIH). Information of antibodies was listed in **Table 1**)

**Table 1:** Antibody information.

Antibody	Company	Catalog	Application(dilution)
Collagen II	Abcam	ab34712	IHC(1:100)/WB(1:500)
MMP13	Abcam	ab39012	IHC(1:100)/WB(1:3000)
p-AMPKα	Cell Signaling Technology	50081S	IHC(1:400)/WB(1:1000)
AMPKα	Cell Signaling Technology	5831S	WB(1:1000)
SIRT1	Abcam	ab110304	IHC(1:200)/WB(1:3000)
LC3B	Cell Signaling Technology	12741S	IHC(1:500)/WB(1:1000)
cleaved caspase-3	Cell Signaling Technology	9664T	IHC(1:2000)/WB(1:1000)
p62	Abcam	ab56416	WB(1:2000)
AMPKα1	Santa Cruz	sc-19128	WB(1:500)
AMPKα2	Proteintech	18167-1-AP	WB(1:1000)
ATG5	Cell Signaling Technology	12994T	WB(1:1000)
BAX	Abcam	ab32503	WB(1:1000)
Bcl2	Abcam	ab182858	WB(1:2000)
GAPDH	Proteintech	60004-1-Ig	WB(1:10000)
AffiniPure Goat Anti-Mouse IgG (H+L)	Jackson ImmunoResearch	115-005-003	WB(1:10000)
AffiniPure Goat Anti-Rabbit IgG (H+L)	Jackson ImmunoResearch	111-005-003	WB(1:10000)

### Terminal Deoxynucleotidyl Transferase-Mediated dTUP-Biotin Nick End Labeling Assay

The chondrocyte apoptosis was detected by transferase-mediated dTUP-biotin nick end labeling assay (TUNEL) assays (Roche, North Carolina, USA). Mouse cartilage tissues were fixed in paraformaldehyde and prepared in paraffin sections. To inactivate endogenous peroxidase activity, 3% H_2_O_2_-methanol solution was used at 37°C for ten minutes. The slides were incubated with 0.2% Triton for five minutes. The samples were incubated with TUNEL reaction mixture according to the manufacturers’ instructions. Nuclei were stained with DAPI (Beyotime, Shanghai, China). A positive sample was carried out in a healthy sample using DNase I (RNase free, Roche, North Carolina, USA), while a negative control sample was performed in samples without a terminal transferase.

### Isolation and Culture of Primary Mice Chondrocytes

Mice articular chondrocytes were isolated from the knee joints of hind limbs of 6~8-week-old 8–10 male C57bl/6 mice (28–30 g), and five independent biological replicates were performed. Individual cell experiments were triplicated as technical replicates. Cartilage was dissected from the joint explant surfaces and then rinsed with saline solution three times. After digesting with collagenase II (1.5 mg/ml Gibco, CA, USA) at 37°C for overnight, the isolated chondrocytes were seeded in flasks. Cells were cultured in Dulbecco’s Modified Eagle Medium Nutrient Mixture F-12 (DMEM/F12, Gibco, CA, USA) with 10% fetal bovine serum (Gibco, CA, USA). Passage 2 cells were seeded in 6-well plates (200,000 cells/well). Metformin (experimental concentration 1mM) was administrated one hour before the stimulation of IL-1β (10 ng/ml). Where indicated, cells were also incubated with 3-MA (an inhibitor of autophagosome formation, purity: 99.97%, 5 mM) (Song et al., 2015), compound C (a selective AMPK inhibitor, purity:99.82%, 10 μM) (Yu et al., 2008), or EX-527(a selective SIRT1 inhibitor, purity:100.0%, 10 μM) (Price et al., 2012) before the experiments.

### Cell Lysis and Western Blot Analysis

The chondrocytes were lysed in cell lysis buffer (25 mM tris-Cl, 250 mM NaCl, 5 mM ethylenediaminetetraacetic acid, 1% CA-630, 1 mM phenylmethylsulphonyl fluoride, 5 mM dithiothreitol, protease and phosphatase inhibitors) for fifteen minutes. A total of 20 μg proteins was loaded in sodium dodecyl sulfate-polyacrylamide gel and transferred to polyvinylidene fluoride membranes. The membranes were further blocked by 5% nonfat milk in tris buffered with Tween-20 for one hour at room temperature and incubated with primary antibodies overnight at 4°C. After incubating with secondary antibodies for one hour at room temperature, the membranes were exposed to chemiluminescence (Epizyme bio, Shanghai, China). The density of each band was detected by Tanon Imager 4600 system (Tanon, Shanghai, China) and quantified by ImageJ software.

### Small Interfering RNA Transfection

Lipofectamine RNAi MAX (Invitrogen, Carlsbad, CA) was used to silence corresponding mRNAs in cultured chondrocytes. Briefly, 125 pmol of small interfering RNA (siRNA) (**Table 2**) and 5 μl of RNAiMax were incubated in Opti-MEM (Gibco, CA, USA) for five minutes. Final concentrations of RNAi (50 nM) were optimized on the preliminary experiments. Cells were seeded in a six-well plate before transfection. After 24 h, cells at 60%–70% confluency were transfected with negative control or specific siRNA duplexes.

**Table 2:** SiRNA information.

Target gene	Sense sequence	Antisense sequence
Scramble	5’-UUC UCC GA CGU GUC ACG UTT-3	5’-ACG UGA CAC GUU CGG AGA ATT-3’
AMPK α1	5’- CUA UGA AUG GAA GGU UGU A-3’	5’-UAC AAC CUU CCA UUC AUA-3’
AMPK α2	5’-GCA GUG GCU UAU CAU CUU ATT-3’	5’-UAA GAU GAU AAG CCA CUG CTT-3’
SIRT1	5′-CCA GUA GCA CUA AUU CCA ATT-3′	5′-UUG GAA UUA GUG CCA CUG GTT-3′
ATG5	5′-GCU ACC CAG AUA ACU UUC UTT-3’	5’-AGA AAG UUA UCU GGG UAG CTT-3’

### Materials

Metformin was bought from Sigma-Aldrich (D150959, purity: 97%, Sigma, St. Louis, USA). Murine IL-1β was bought from Preprotech (211-11B, purity: ≥98%, Preprotech, Chicago, USA). Compound C (S7840, purity: 99.82%) and 3-MA (S2767, purity: 99.97%) were bought from Selleck (Shanghai, China), EX-527 was bought from Beyotime (SC0281, purity:100.0%, Beyotime, Shanghai, China). Antibodies used in Western Blot and IHC were listed in **Table 1**.

### Statistical Analysis

Data were expressed as the means ± standard deviation (SD). The data were first analyzed for normal distribution. A comparison between two groups was performed using two-tailed Student’s t-test. Comparisons of more than two groups were performed using analysis of variance (ANOVA) followed by Tukey’s test, as appropriate. Null-hypotheses were that no difference between experimental groups and control group or no difference between experimental groups, as appropriate. Statistical analysis was performed using GraphPad Prism 7.0 (GraphPad Software Inc., San Diego, CA) and SPSS 19.0 (IBM, Armonk, NY, USA). Differences with P values of less than 0.05 were considered statistically significant.

## Results

### Metformin Attenuates Cartilage Degradation in Mice With OA

**Figure 1:**
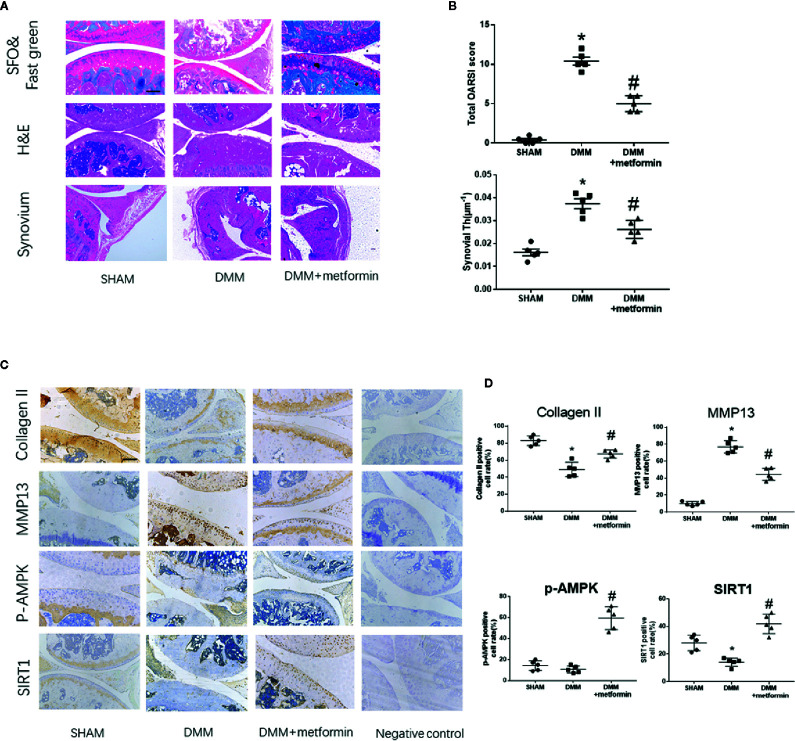
Metformin treatment attenuates osteoarthritis **(A)** Representative H&E staining, S-O Fast Green staining and synovium region of knee joint samples from different experimental groups at 8 weeks post-surgery (scale bar: 200 μm). White arrow: Regional proteoglycan loss **(B)** OARSI scoring system and synovial thickness measurement were applied for histopathological evaluation **(C)** IHC analysis of type II collagen, MMP13, p-AMPK, SIRT1 expression in mouse knee medial femoral condyle and medial tibial plateau cartilage, the forth lanes are negative controls (scale bar:200μm). **(D)** The percentage of positive cells according to immunohistochemistry. The data were shown as means ± SD. *P < 0.05, compared with sham group, ^#^P < 0.05(n=5) compared with destabilization of the medial meniscus (DMM) groups. H&E, hematoxylin and eosin; S-O, Safranin O; IHC, Immunohistochemistry; MMP13, Matrix Metallopeptidase 13; p-AMPK, Phospho- 5’ adenosine monophosphate-activated protein kinase; SIRT1, silent mating type information regulation 2 homolog1; OARSI, Osteoarthritis Research Society International; SD, standard deviation.

There were no structural changes at the anterior cruciate ligament or meniscus in the knees of the sham group. Both Safranin-O staining and OARSI scores indicted that the surgery induced severer articular cartilage damage and increased the thickness of the synovial membrane compared with the sham group. Regional proteoglycan loss and vertical clefts or erosion occurred in both medial tibial plateau (MTP) and medial femoral condyle (MFC) 8-week after the surgery (**Figure 1A**, white arrow). Compared with the DMM mice, intraarticular metformin administration effectively alleviated the cartilage destruction, increased the proteoglycan expression in Safranin O-fast green staining, and significantly reduced the thickness of the synovial membrane (**Figures 1A, B**).

Immunohistochemical staining showed that the surgery induced an increased expression of matrix metallopeptidase 13 (MMP13) and decreased expressions of type II collagen and SIRT1 in the articular cartilage. Phosphorylated levels at Thr172 residue of AMPKα were comparable in control and DMM surgery groups (**Figures 1C, D**). Metformin treatment reduced the protein expression of MMP13 and increased type II collagen protein expression in mouse articular cartilage tissues compared with the DMM surgery group. Metformin significantly increased the phosphorylated level of AMPKα as well as SIRT1 protein expression in the knee joint of mice (**Figures 1C, D**). These results collectively show that the intraarticular injection of metformin mitigated articular cartilage degradation, shown as an increase in type II collagen and a decrease of MMP13, with concomitant activation of AMPK and SIRT1.

### Metformin Activates Autophagy and Reduces Apoptosis in Mice With OA

**Figure 2:**
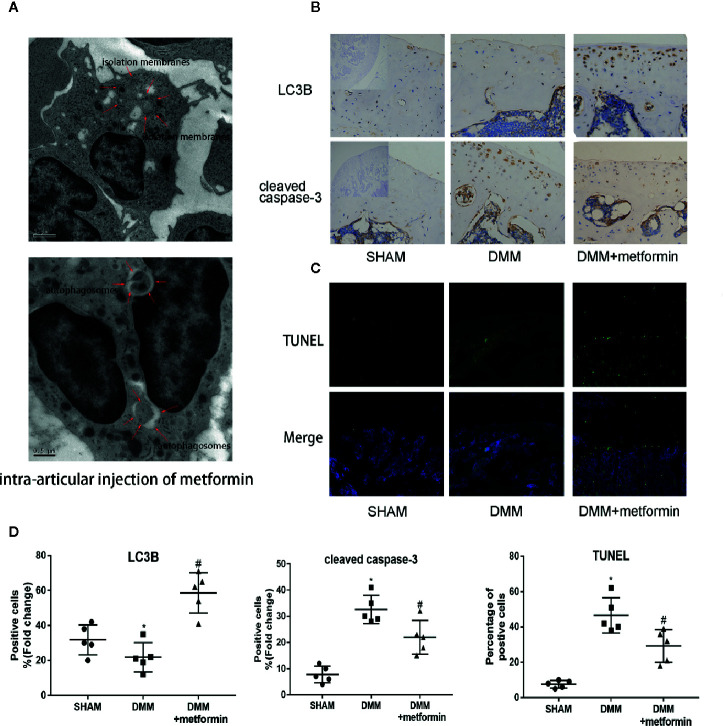
Metformin activates autophagy and reduces apoptosis in mice of DMM model **(A)** TEM images of the articular cartilage tissues from mice with intraarticular injection of metformin. Red arrows indicate the autophagy bodies of chondrocytes. **(B)** Immunohistochemistry of LC3B and cleaved caspase-3, the top left of the first lanes are negative controls (scale bar: 100μm). **(C)** TUNEL staining of knee joint samples eight weeks after the surgery. (scale bar:100μm). **(D)** The percentage of positive cells, according to immunohistochemistry. Changes in the percentage of TUNEL-positive cells in DAPI-positive cells are shown. Significant differences between the DMM and SHAM groups are indicated as *P < 0.05, Significant differences between metformin group and DMM group are indicated as ^#^P < 0.05(n=5). DMM, destabilization of the medial meniscus; TEM, Transmission electron microscope; LC3B, Microtubule-associated proteins 1A/1B light chain 3B; TUNEL, Terminal deoxynucleotidyl transferase dUTP nick end labeling; DAPI, 4′,6-diamidino-2-phenylindole.

After the surgery, ultrastructural analysis by TEM observed autophagic bodies in the cartilage of mice treated with metformin (**Figure 2A**). Consistently, decreased LC3B signals in the IHC examination were observed in cartilage. The presence of LC3B protein in the cartilage was significantly increased in mice treated with metformin (**Figure 2B**).

Presences of cleaved caspase-3 in IHC examination and TUNEL signal were significantly enhanced in the cartilage of the surgery group, compared with the control. Metformin reduced the expression of cleaved caspase‐3 and the TUNEL signals in the cartilage tissue (**Figures 2C, D**). These results collectively show that the administration of metformin activated cellular autophagy and decreased chondrocyte apoptosis.

### Metformin Activates AMPK/SIRT1-Mediated Autophagy in the Primary Cultured Chondrocytes

**Figure 3:**
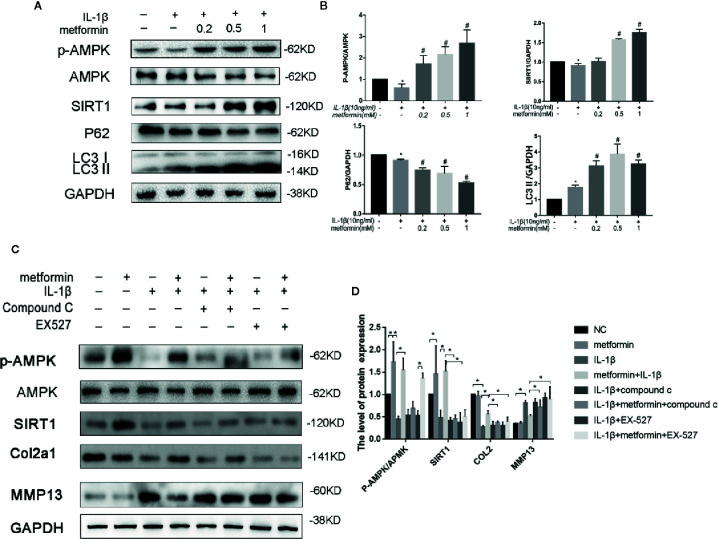
Metformin activates AMPK/SIRT1-mediated autophagy in cultured chondrocytes. Representative Western blots **(A)** and densitometric quantification **(B)** of p-AMPKα, AMPKα, SIRT1, p62, and LC3B in the chondrocytes treated with different concentration of metformin in the presence of IL-1β. Expressions of target protein were normalized to GAPDH and shown as means ± SD. *P < 0.05 compared to control group, ^#^P < 0.05 compared to the cells treated with IL-1β (n=5). Representative Western blots **(C)** and densitometric quantification **(D)** of p-AMPKα, AMPKα, SIRT1, Collagen II, MMP13 treated with AMPK inhibitor Compound C or SIRT1 inhibitor EX527 in IL-1β-stimulated (10ng/ml) chondrocytes with or without metformin (1mM) for 24h. Expressions of target protein were normalized to GAPDH and shown as means ± SD. *P < 0.05 compared to control group or as indicated (n=5). AMPK, 5’ adenosine monophosphate-activated protein kinase; SIRT1, silent mating type information regulation 2 homolog1; p-AMPK, Phospho- 5’ adenosine monophosphate-activated protein kinase; p62, Sequestosome-1; LC3B, Microtubule-associated proteins 1A/1B light chain 3B; MMP13, Matrix Metallopeptidase 13; EX527, Selisistat; IL, Interleukin; GAPDH, Glyceraldehyde 3-phosphate dehydrogenase; SD, standard deviation.

Murine chondrocytes were isolated and cultured *in vitro*. Interleukin 1 beta (IL-1β) (10 ng/ml), a proinflammatory cytokine in OA (Towle et al., 1997), reduced Sequestosome-1(p62) protein expression and increased LC3II expression. IL-1β significantly reduced phosphorylated levels of AMPKα at Thr172 residue, but not total AMPKα protein. IL-1β downregulated SIRT1 protein expression as well (**Figures 3A, B**). Metformin increased the expression of LC3II and decreased the expression of p62 in cells challenged with IL-1β. Consistently metformin treatment significantly increased the phosphorylated level of AMPK, upregulated SIRT1 and LC3II proteins, and downregulated p62 protein in a dose-dependent fashion (**Figures 3A, B**). IL-1β stimulation significantly increased expressions of MMP13 protein and decreased expressions of type II collagen protein in cultured chondrocytes (**Figures 3C, D**).

In the presence of IL-1β, metformin reduced the protein expression of MMP13 and increased the protein expression of type II collagen (**Figures 3C, D**).

In the presence of metformin and IL-1β, compound C significantly reduced the phosphorylation of AMPK and decreased the expression of SIRT1. Compound C incubation significantly reduced type II collagen expression and increased MMP13 expression. (**Figures 3C, D**).

In the presence of metformin and IL-1β, Selisistat (EX-527) pretreatment significantly inhibited the expression of SIRT1 compared with the metformin group. EX-527 incubation significantly reduced type II collagen expression and increased MMP13 expression as well (**Figures 3C, D**).

**Figure 4:**
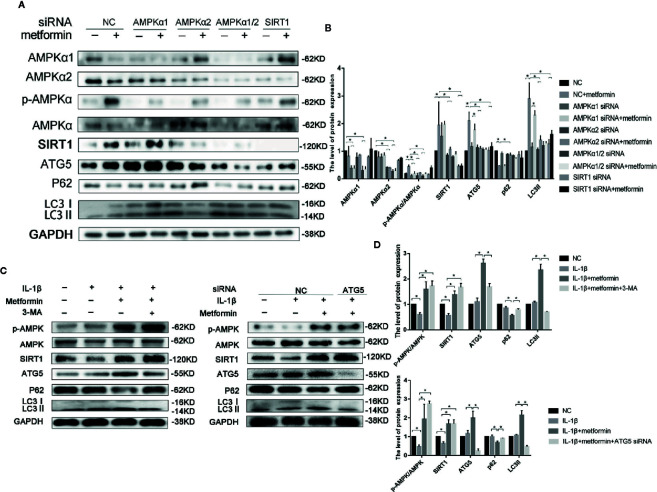
Metformin activates autophagy through AMPK/SIRT1 pathway. Representative Western blots **(A)** and densitometric quantification **(B)** of AMPKα1, AMPKα2, p-AMPKα, AMPKα, SIRT1, ATG5, p62, LC3 in the chondrocytes transfected with negative control siRNA (con-siRNA) or AMPKα1/2 or SIRT1 siRNA with or without metformin. Expressions of target protein were normalized to GAPDH and shown as means ± SD. *P < 0.05 compared to control or unrelated siRNA or as indicated. Representative Western blots **(C)** and densitometric quantification **(D)** of p-AMPK(Thr172), AMPKα, SIRT1, ATG5, p62 and LC3 in IL-1β-stimulated chondrocytes incubated with or without metformin in the absence or presence of 3-MA (10 mM) or ATG5 siRNA for 24 h. Expressions of target protein were normalized to GAPDH and shown as means ± SD. Significant differences between the treatment and control groups are indicated as *P<0.05 compared to control or IL-1β or IL-1β plus metformin. AMPK, 5’ adenosine monophosphate-activated protein kinase; SIRT1, silent mating type information regulation 2 homolog1; ATG5, Autophagy related 5; p62, Sequestosome-1; LC3, Microtubule-associated proteins 1A/1B light chain 3; siRNA, small interfering RNA; SD, standard deviation; 3-MA, 3-Methyladenine; GAPDH, Glyceraldehyde 3-phosphate dehydrogenase; IL, Interleukin.

To further explore the role of AMPKα/SIRT1 in autophagy signaling, small-interfering RNA of AMPKα1/2 or SIRT1 was used in the present study. AMPKα1/2 siRNA transfection diminished metformin-induced phosphorylation of AMPK. Silencing AMPKα2, but not AMPKα1, markedly reduced protein expressions of LC3-II and autophagy-related 5 protein (ATG5), and increased the expression of p62 in cells treated with IL-1β and metformin. AMPKα2 siRNA, but not AMPKα1, significantly decreased SIRT1 expression as well. Silencing SIRT1 abolished metformin-exerted protective effects on the activation of autophagy as well (**Figures 4A, B**).

ATG5 siRNA significantly decreased the ATG5 expression in chondrocytes. Incubation of 3-MA and ATG5 siRNA significantly decreased the level of LC3II and increased the expression of p62 in cells incubated with IL-1β and metformin. However, inhibition of autophagy failed to affect the phosphorylated level of AMPKα and SIRT1 expressions (**Figures 4C, D**).

**Figure 5:**
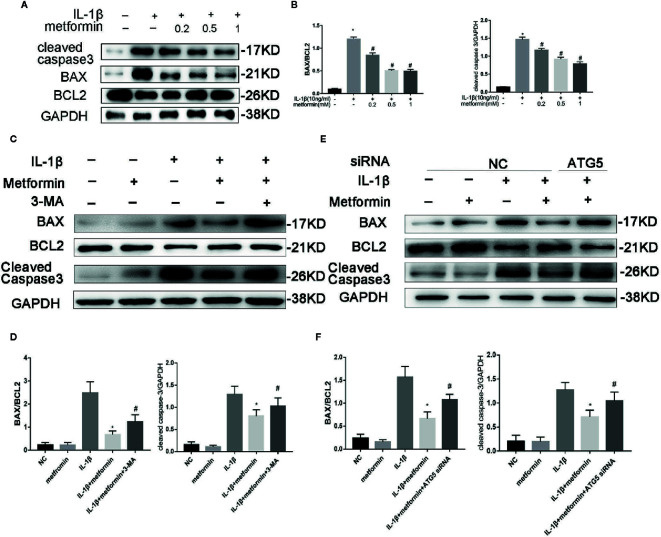
Metformin protects chondrocytes against IL‐1β-induced apoptosis. Representative Western blots **(A)** and densitometric quantification **(B)** of BAX, Bcl2 and Cleaved Caspase-3 in cultured chondrocytes treated with IL-1β. Representative Western blots **(C)** and densitometric quantification **(D)** of BAX, Bcl2 and Cleaved Caspase-3 in chondrocytes incubated with or without metformin and IL-1β after 3-MA pretreatment. Representative Western blots **(E)** and densitometric quantification **(F)** in cultured chondrocytes when cells are incubated with or without metformin and IL-1β after ATG5 siRNA transfection. Expressions of target protein were normalized to GAPDH and shown as means ± SD. Significant differences between the treatment and control groups are indicated as *P<0.05 compared to group IL-1β; ^#^P < 0.05 compared to the cells treated with IL-1β plus metformin(n=5). IL, Interleukin; BAX, BCL2(B-cell lymphoma 2) Associated X; 3-MA, 3-Methyladenine; ATG5, Autophagy related 5; siRNA, small interfering RNA; GAPDH, Glyceraldehyde 3-phosphate dehydrogenase; SD, standard deviation.

Of the known regulators of apoptosis, the best characterized is the Bax(B-cell lymphoma 2 Associated X) to Bcl-2(B-cell lymphoma 2) ratio (Raisova et al., 2001) and the level of cleaved caspase 3 (Porter and Jänicke, 1999). IL‐1β stimulation increased the protein expression of cleaved caspase-3 and the ratio of BAX/Bcl2. Metformin significantly decreased the expression of cleaved caspase-3 and the ratio of BAX/Bcl2 in the presence of IL-1β (**Figures 5A, B**).

In the presence of metformin, 3-MA or ATG5 siRNA increased cleaved caspase-3 expression and the ratio of BAX/Bcl2 protein. (**Figures 5C–F**).

## Discussion

The present study demonstrates that metformin alleviates OA by promoting autophagy and inhibiting apoptosis in a mouse DMM model. The metformin-exerted protective effect is through activating the AMPKα2/SIRT1 pathway.

In the present study, destabilization of the medial meniscus surgery increases the expression of MMP13 and decreases expressions of type II collagen and proteoglycans, indicating that the surgery successfully induces OA-like changes in the knee. IL-1β increases metalloproteinases expressions and induces apoptosis in cultured chondrocytes, supporting the note that IL-1β is an important inflammatory cytokine in the process of OA (Jenei-Lanzl et al., 2019; Zhao et al., 2019).

Autophagy is a homeostatic process adjusting cell metabolic status on nutrient supply and removing damaged macromolecules and organelles (Mizushima, 2009). In the present study, blunt autophagy is observed in the knee of DMM mice, confirming the impairment of autophagy in the development of OA (Lotz and Carames, 2011). AMPK/SIRT1 axis is the upstream signaling for autophagy (Morselli et al., 2010; Kim et al., 2011). AMPK-activated autophagy protects against lung fibrosis (Rangarajan et al., 2018), hepatic steatosis (Song et al., 2015), and intervertebral disc degeneration (Chen et al., 2016). Activation of AMPKα protein, shown as phosphorylation at Thr 172 residue (Hawley et al., 1996), mitigates IL-1β-induced catabolic gene expression in chondrocytes (Terkeltaub et al., 2011). Both AMPKα1 and α2 subunits take part in the activation of AMPK. It is reported that conditional knockout of AMPKα1 and/or α2 in chondrocytes accelerates the progress of OA in a mouse model (Zhou et al., 2017). Nevertheless, specific deletion of AMPKα1 in chondrocytes does not affect the process of arthritis in mice, which is probably due to a compensatory upregulation of AMPKα2 (Yang et al., 2016). In the present study, silencing AMPKα2, but not AMPKα1, prevents autophagy in chondrocytes, implying that AMPKα2 is the active subunit for AMPK activation.

SIRT1 plays a crucial role in tissue homeostasis, including cartilage (Yeung et al., 2004). SIRT1 protein expression is diminished in the region of OA (Fujita et al., 2011) Mechanical stress accelerates the progress of OA in cartilage-specific SIRT1 knockout mice (Matsuzaki et al., 2014). AMPK regulates SIRT1 expression in skeletal muscle (Iwabu et al., 2010) and adipocytes (Chau et al., 2010). In the present study, inhibition of AMPK by compound C decreases SIRT1 protein expression, while blockade of SIRT1 by EX-527 does not affect AMPKα activation, indicating that the AMPK pathway is required for SIRT1 activation. Inhibition of AMPK/SIRT1/autophagy by either pharmacological agents or genetic approach augment OA phenotype, further supporting the note that activation of AMPK/SIRT1 signaling is vital in maintaining cartilage homeostasis.

**Figure 6:**
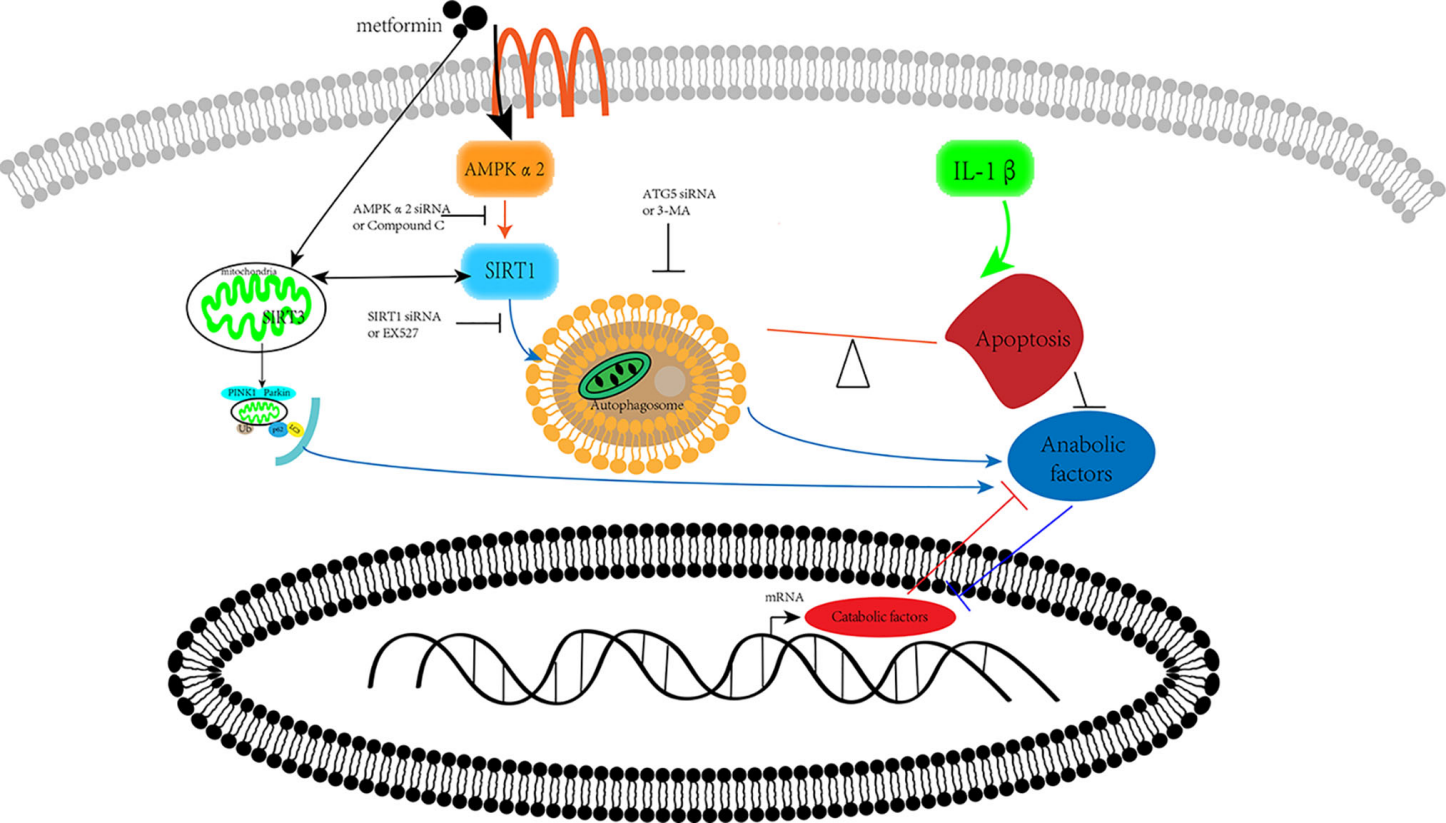
Schematic illustration. Metformin prevents cartilage degradation in a mouse model of osteoarthritis. Metformin activates AMPKα2/SIRT1 pathway, upregulates autophagy, and downregulates apoptosis in articular chondrocytes through the AMPK/SIRT1 pathway. AMPK, 5’ adenosine monophosphate-activated protein kinase; SIRT1, silent mating type information regulation 2 homolog1.

Our previous research has shown that metformin exerts protective effects *via* SIRT3 activated PINK1/Parkin-mediated-mitophagy in cultured chondrocytes (Wang et al., 2019). In human brain microvascular endothelial cells and astrocytes, Sirt1 regulated Sirt3 expression *via* the AMPK-PGC1 pathway in oxygen and glucose deprivation model (Chen et al., 2018). Thus, we assumed that these two SIRT proteins have a synergic effect on the protection of chondrocytes from inflammation (**Figure 6**).

Metformin treatment reduces cell apoptosis in cultured chondrocytes and mice keens with OA, suggesting that metformin exerts protective effects against cell apoptosis. In the present study, inhibition of autophagy, by either pharmacological or genetic approach, reverses the protective effects of metformin, confirming the critical role of maintaining the balance between apoptosis and autophagy. It further indicates that the protective effects of metformin in the process of OA are attributed to promoting autophagy and diminishing apoptosis. It is reported that autophagy protein Atg4B bounds to apoptotic protein Bcl-2, leading to a switch from apoptosis to autophagy in A549 cells (Li et al., 2019). By targeting phorbol-12-myristate-13-acetate-induced protein 1, a member of the pro-apoptotic B-cell lymphoma 2 family (also known as Noxa), autophagy regulates apoptosis through its degradation (Wang et al., 2018). In the present study, inhibition of autophagy potentiates the occurrence of apoptosis, confirming the interaction between apoptosis and autophagy.

There are some limitations to the present study. We did not use human samples to evaluate the potential therapeutic effect of metformin for human OA. Since clinical studies regarding the protective effects of metformin on OA are inconclusive. Further experiment could be conducted to evaluate the protective effect of metformin in human cartilage sample.

The present study reports that metformin alleviates DMM-induced OA-like change in mice knee joint through activating autophagy and downregulating apoptosis (**Figure 6**). Metformin exerts its protective effects against OA through the AMPKα2/SIRT1 pathway. These findings shed light on the therapeutic potential of metformin in the treatment of OA.

## Data Availability Statement

The raw data supporting the conclusions of this article will be made available by the authors, without undue reservation.

## Ethics Statement

The animal study was reviewed and approved by Ethics Committee for Animal Research (Zhongshan Hospital, Shanghai, China).

## Author Contributions

All listed authors have made substantial contributions to the following aspects of the manuscript: (1) The conception and design of the study, or acquisition of data, or analysis and interpretation of data. (2) Drafting the article or revising it critically for important intellectual content. (3) Final approval of the version to be submitted.

## Funding

This work was supported by the National Natural Science Foundation of China (Grant number 81971308).

## Conflict of Interest

The authors declare that the research was conducted in the absence of any commercial or financial relationships that could be construed as a potential conflict of interest.

## Supplementary Material

Supplementary Figure 1 H&E staining and S-O Fast Green staining of knee joint samples from other mice of different experimental groups at 8 weeks post-surgery with or without metformin treatment (scale bar: 200 μm). H&E, hematoxylin and eosin; S-O, Safranin O.

Supplementary Figure 2 IHC of type II collagen, MMP13, p-AMPK, SIRT1 expression in mouse knee joint cartilage from other mice of different experimental groups at 8 weeks post-surgery with or without metformin treatment (scale bar:100μm). IHC, Immunohistochemistry; MMP13, Matrix Metallopeptidase 13; p-AMPK, Phospho- 5’ adenosine monophosphate-activated protein kinase; SIRT1, silent mating type information regulation 2 homolog1.

## Abbreviations

AMPK, 5’ adenosine monophosphate-activated protein kinase; ATG: autophagy related; BAX, BCL2(B-cell lymphoma 2) Associated X; ECM, extracellular matrix; EX527, Selisistat; GAPDH, Glyceraldehyde 3-phosphate dehydrogenase; H&E, hematoxylin and eosin; IHC, Immunohistochemistry; IL, Interleukin; LC3B: microtubule associated protein 1 light chain 3 beta; MMP13, Matrix Metallopeptidase 13; p62: sequestosome 1; S-O, Safranin O; OARSI, Osteoarthritis Research Society International; OA, Osteoarthritis; p-AMPK, Phospho-5’ adenosine monophosphate-activated protein kinase; siRNA, small interfering RNA; SIRT1, silent mating type information regulation 2 homolog1; TEM, Transmission electron microscope; TUNEL: terminal deoxynucleotidyl transferase-mediated dUTP nick end labeling; 3-MA, 3-Methyladenine.
