# Supplementary figures and images for: Metformin Mitigates Cartilage Degradation by Activating AMPK/SIRT1-Mediated Autophagy in a Mouse Osteoarthritis Model
Source: Front Pharmacol. 2020 Jul 24;11:1114. doi: 10.3389/fphar.2020.01114 (PMC7393141; doi:10.3389/fphar.2020.01114)

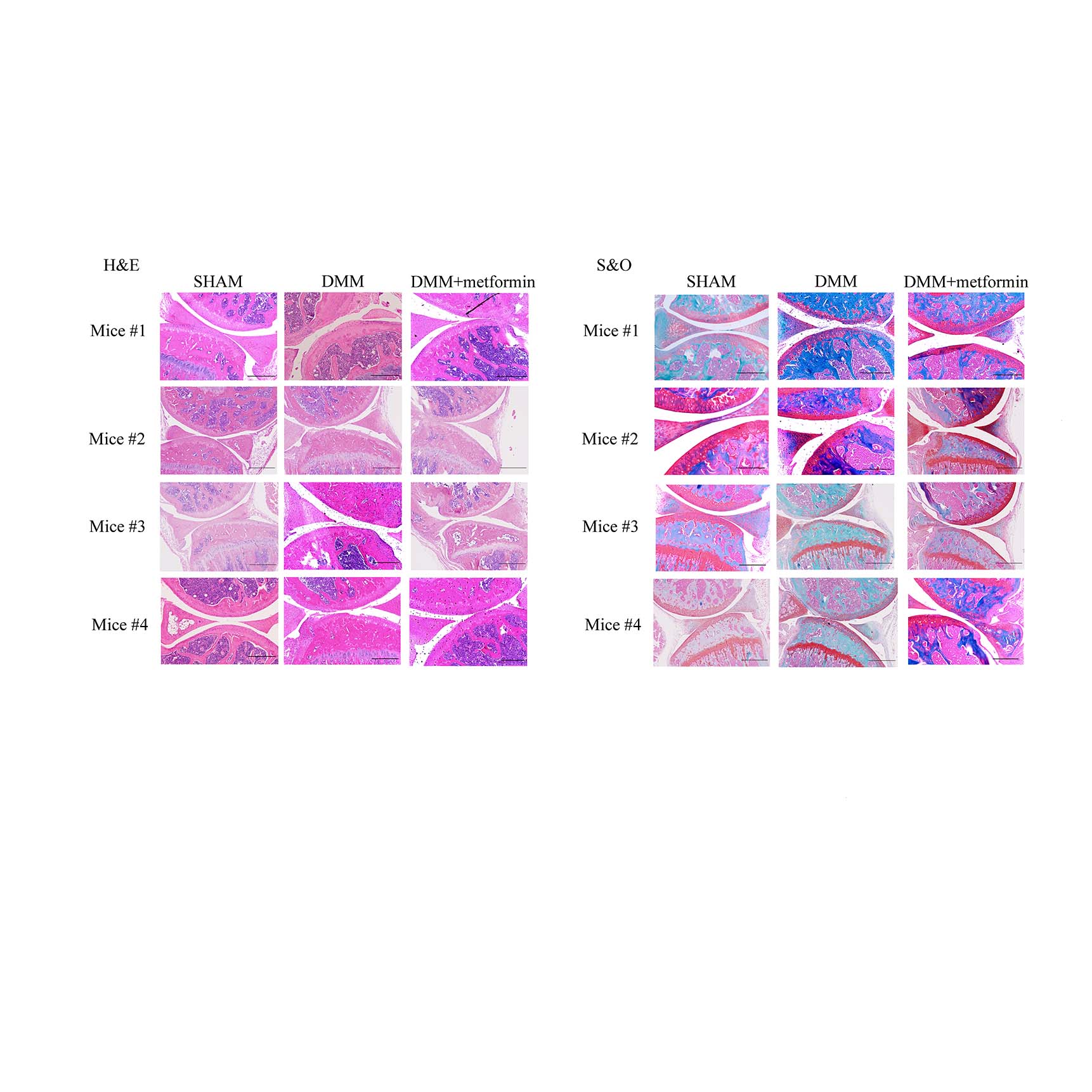

Supplement: Supplementary Figure 1 — H&E staining and S-O Fast Green staining of knee joint samples from other mice of different experimental groups at 8 weeks post-surgery with or without metformin treatment (scale bar: 200 μm). H&E, hematoxylin and eosin; S-O, Safranin O. [file Image_1.jpeg]

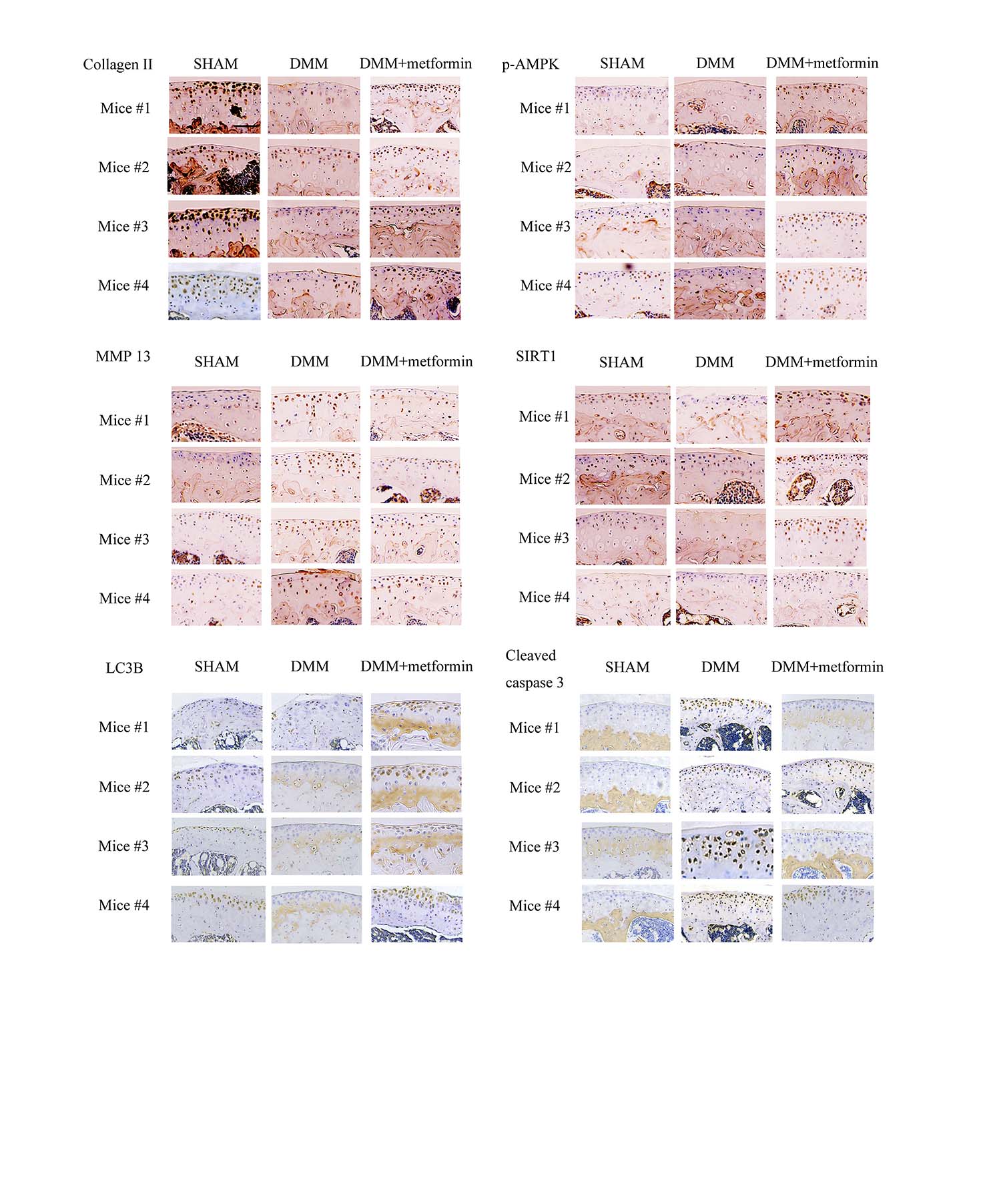

Supplement: Supplementary Figure 2 — IHC of type II collagen, MMP13, p-AMPK, SIRT1 expression in mouse knee joint cartilage from other mice of different experimental groups at 8 weeks post-surgery with or without metformin treatment (scale bar:100μm). IHC, Immunohistochemistry; MMP13, Matrix Metallopeptidase 13; p-AMPK, Phospho- 5’ adenosine monophosphate-activated protein kinase; SIRT1, silent mating type information regulation 2 homolog1. [file Image_2.jpeg]
